# Evaluating and implementing machine learning models for personalised mobile health app recommendations

**DOI:** 10.1371/journal.pone.0319828

**Published:** 2025-03-19

**Authors:** Hafsat Morenigbade, Tareq Al Jaber, Neil Gordon, Gregory Eke

**Affiliations:** 1 Centre of Excellence for Data Science, AI and Modelling, Faculty of Science and Engineering, University of Hull, United Kingdom; 2 School of Computer Science, Faculty of Science and Engineering, University of Hull, United Kingdom; BRAC Business School, BRAC University, BANGLADESH

## Abstract

This paper focuses on the evaluation and recommendation of healthcare applications in the mHealth field. The increase in the use of health applications, supported by an expanding mHealth market, highlights the importance of this research. In this study, a data set including app descriptions, ratings, reviews, and other relevant attributes from various health app platforms was selected. The main goal was to design a recommendation system that leverages app attributes, especially descriptions, to provide users with relevant contextual suggestions. A comprehensive pre-processing regime was carried out, including one-hot encoding, standardisation, and feature engineering. The feature, “Rating_Reviews”, was introduced to capture the cumulative influence of ratings and reviews. The variable ‘Category’ was chosen as a target to discern different health contexts such as ‘Weight loss’ and ‘Medical’. Various machine learning and deep learning models were evaluated, from the baseline Random Forest Classifier to the sophisticated BERT model. The results highlighted the efficiency of transfer learning, especially BERT, which achieved an accuracy of approximately 90% after hyperparameter tuning. A final recommendation system was designed, which uses cosine similarity to rank apps based on their relevance to user queries.

## 1. Introduction and background

mHealth applications have become increasingly popular due to smartphones and their widespread accessibility. These apps have become widely available, portable, and adaptable tools for a variety of health-related tasks as a result of the widespread use of smartphones and advanced mobile technologies. These apps have removed barriers to accessing healthcare and revolutionized patient self-management, covering everything from telemedicine consultations to fitness tracking, and prescription reminders to chronic disease management. Their growth is reflected in the projected CAGR (compound annual growth rate) of 21.1% from 2019-2026 [1], signifying their important role in modern healthcare.

Although mHealth apps offer many benefits, their abundance has created a conundrum of choice. Users often find it difficult to select an app that suits their health needs among thousands of alternatives. The current lack of standardisation also raises issues of efficacy, safety, and confidentiality. Despite some commendable efforts, such as the Mobile App Rating Scale (MARS) created by Stoyanov et al. (2016) [2], to classify and evaluate health applications, these usually revolve around general quality parameters. The mHealth landscape still lacks a personalised, condition-specific recommendation system. Recent studies have highlighted the need for more sophisticated analysis and recommendation systems. For instance, He et al. (2024) explored factors influencing user satisfaction in hypertension management apps, revealing the asymmetrical nature of user reviews [3]. Similarly, Wang et al. (2022) used machine learning to analyse user-generated content from weight loss apps, identifying key factors correlated with user satisfaction [4]. These studies underscore the importance of tailored app recommendations that consider specific user needs and preferences.

Given the complexity of the mHealth landscape, a robust classification system is necessary to analyse the magnitude of apps on the market and suggest which ones are most appropriate for different health conditions. A solution like this would make it easier for consumers to choose apps and ensure they have useful options. Addressing this gap would lead to informed choices, thus enhancing the potential benefits of mHealth. Given the rapid digitalisation of healthcare and the increasing reliance on personal health management systems, this research is highly relevant and significant. To achieve a more comprehensive and patient-centred approach to care, a successful classification system would pave the way for the integration of mHealth app recommendations into bigger healthcare systems and Electronic Health Records (EHRs).

Park et al. (2020) discussed the dynamics of service quality attributes in different contexts, emphasizing the role of satisfiers and dissatisfiers [5]. This concept is crucial for understanding how users perceive and engage with health apps, which informs the development of effective recommendation systems.

The sole and overarching goal of this study is to evaluate various machine learning models to identify the best model for recommending personalized mobile health apps based on their relevance to specific health conditions. Subsequently, the selected model was implemented in a Jupyter Notebook to create an interactive model implementation that accepts input and produces accurate output. This was only done to cross validate and ensure the model’s effectiveness. This study aims to develop a module that provides personalised, pertinent, and trustworthy health app suggestions by drawing on a large dataset of app features, user demographics, and health status information.

In the rapidly evolving landscape of mHealth apps, it is vital to understand the factors driving user engagement and to identify methods that elevate the overall user experience. Furthermore, pinpointing strategies that not only ensure the relevance and quality of recommended apps but also contribute positively to health outcomes is essential. With these considerations in mind, this study proposes a classification module with objectives to:

- Evaluate and identify the best machine learning model for recommending personalized mobile health apps.
- Cross validate model accuracy by implementing the selected model in an interactive application to provide accurate and personalized app suggestions.
- Enhance the overall mHealth app experience by ensuring the recommendations are relevant and beneficial to users’ health outcomes.
- Promote positive health outcomes through app recommendations.

Essentially, this research strives to combine the fields of technology and healthcare, creating a solution that meets the demands of the contemporary health-conscious person.

The field of mHealth applications has seen multiple attempts at creating classification systems. Keyword-based methods remain a popular approach, where apps are ranked based on user-generated keywords or those provided by the app developers themselves [6]. While this method is simple, it often overlooks apps that fit a certain niche or those that don’t use a widely understood language. Furthermore, it ignores the effectiveness, relevance, and quality of apps in favour of their more superficial features. User review aggregations offer another popular approach to categorising mHealth apps. Apps are ranked based on average user ratings, with users often choosing apps with higher ratings [7]. However, this method tends to favour well-known apps and the classification is determined more by the number of users than the inherent value of the app or applicability to health conditions.

The Mobile App Rating Scale (MARS), developed by Stoyanov et al. (2016) [2], presents a more holistic approach. MARS rates the quality of health apps based on specific factors such as engagement, functionality, aesthetics, and information quality. While this approach presents a step in the right direction, it lacks the aspect of personalised health condition-based recommendations. Despite these advances, a clear gap remains in the current literature: the lack of a comprehensive recommendation system tailored to individual health conditions. Existing systems focus primarily on generalised criteria such as popularity, user ratings, and broad-spectrum quality factors, which do not consider individual health needs in their evaluations [8].

The importance of the mHealth market is evident in its growth statistics. According to a Global mHealth Market (2020) report, the field is expected to grow to $189.3 billion by 2025, up from $40.7 billion in 2020 [1]. This exponential growth is indicative of increasing confidence in these tools for healthcare solutions.

## 2. Methodology

### 2.1. Data collection

The foundational step of this research was to obtain a comprehensive dataset, essential for further analysis. Google Play Store, due to its wide range of app data and accessibility, was the primary choice for the data source. Using web scraping, a technique for collecting large amounts of data from websites, the Beautiful Soup Python library was engaged to extract data from mobile health apps. This method required dissecting the structures of webpages to retrieve the desired information.

The “Inspect” tool, a feature available in most web browsers (e.g., Google Chrome), allows users to examine and interact with the HTML and CSS of a web page. By right-clicking on a webpage and selecting “Inspect,” users can access the underlying code structure, which helps in identifying and extracting relevant app details such as app names, categories, ratings, and descriptions. This tool was crucial in dissecting the Play Store’s webpage structure, enabling precise data extraction.

To screen mobile health applications from the Google Play Store, the following criteria were employed: Apps were initially filtered by selecting categories related to health and fitness, such as “Health & Fitness” and “Medical.” Within these broad categories, apps were further screened based on keywords in their names and descriptions to identify specific health functions (e.g., “Sleep,” “Pregnancy,” “Diabetes”).

The decision to use only the Google Play Store was due to several factors. Firstly, the Google Play Store offers a wide and accessible range of app data. Secondly, Apple’s App Store presents challenges for data extraction as Apple does not readily provide their data and the web scraping tools were ineffective when attempted on their platform.

This endeavour resulted in a collection of data from 894 health apps, predominantly in the “Health & Fitness” and “Medical” categories. Essential features like App Name, Category, and Rating were meticulously extracted. After extraction, the data was structured and stored in CSV format, which streamlined subsequent processing and analysis steps.

### 2.2. Data cleaning and preprocessing

Upon preliminary inspection of the dataset, the apps were broadly categorized into “Health & Fitness” and “Medical.” To better understand the specific functionalities of health apps, a keyword-based approach was used. The apps were segmented into nuanced health categories based on keywords in their names, like “Sleep” or “Pregnancy,” refining them under labels like “SLEEP” or “FERTILITY”, and other key words such as “Therapy”, “Stress”, “Relax” and “Anxiety” were classified under “Mental Wellbeing”. This segmentation was key to ensuring that users could easily identify the right apps tailored to their distinct healthcare needs. Broad categories could potentially obscure app discovery, making it difficult for users to identify the right solution. The new categorization has streamlined the application selection process.

The scientific basis for this classification system is supported by established health informatics frameworks that emphasize the need for categorizing health technologies based on user-centric parameters such as functionality, user engagement, and health outcomes. For instance, Zapata et al. (2015) [6] conducted a systematic literature review on the usability of mHealth apps, highlighting the importance of categorizing apps based on their specific health functionalities and user needs to improve usability and effectiveness [6]. This methodical approach supports the development of a personalized recommendation system by providing a structured dataset that captures the nuances of various health app functionalities.

A thorough completeness check unveiled that some apps were missing ratings. Out of the 894 apps, 314 were missing ratings, possibly due to the absence of user feedback on the Play Store. Additionally, 94 entries in the ‘Size’ column were listed as ‘Varies with device,’ making it difficult to standardize this data. To maintain analytical accuracy, a dual strategy was adopted: firstly, an ‘is_unrated’ column was introduced to track apps initially without ratings. Secondly, the missing ratings were filled using the mean rating of the existing data. This method ensured unbiased data representation by preventing analytical skewness that could arise from excluding unrated apps or assigning them arbitrary ratings.

For the ‘Size’ column, a new column ‘Size_numeric’ was created to standardize the size data. The size values in kilobytes (k) were converted to megabytes by dividing by 1024, and then all the data in the ‘Size’ column in megabytes (M) were converted to numeric form directly. For entries listed as ‘Varies with device,’ the ‘Size_numeric’ column was populated with the mean size value calculated from the available data. This approach ensured a consistent representation of app sizes across the dataset.

Furthermore, to enhance data clarity, non-essential columns, such as ‘Current Ver’, ‘Android Ver’, ‘Type’, ‘Genres’, were discarded. This focus on essential data ensured that the analysis remained relevant and streamlined.

The scientific basis for using average values to fill missing data is supported by data imputation techniques commonly used in statistical analysis. These techniques help to maintain the integrity of the dataset by preventing the loss of valuable information that could arise from simply excluding incomplete entries. According to Little and Rubin (2019) [9], imputing missing data with the mean is a widely accepted method that can provide robust results when the proportion of missing data is relatively small and the data is missing at random [9].

### 2.3. Exploratory Data Analysis (EDA)

#### 2.3.1. Category Distribution.

The exploratory data analysis began by examining the distribution of categories within the dataset. A count plot was employed using the Seaborn library to visualize the distribution of health categories across the apps in our dataset.

**Fig 1:**
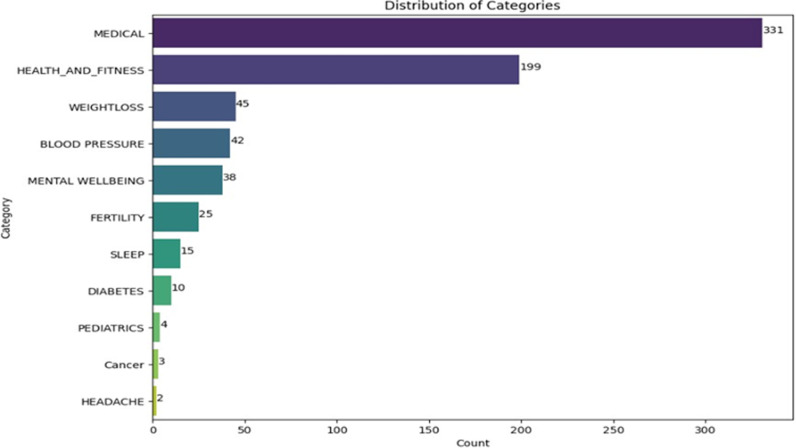
Distribution showing the count of each mHealth category.

In Fig 1, the EDA focused on understanding the distribution of health app categories in the dataset. Using a count plot visualization, it was observed that Medical and Health_and_fitness dominates, with counts of 331 and 199, respectively. However, as the analysis focused on specific health concerns, a disparity in app representation was evident. For instance, niche areas like Paediatrics, Cancer, and Headache had fewer apps, respectively at 4, 3, and 2, respectively. This gap in distribution suggests that while general health apps are abundant, there might be potential market gaps in specialized health areas.

#### 2.3.2. App ratings distribution.

An analysis of the distribution of ratings provides insight into user reception and satisfaction with the health apps.

**Fig 2:**
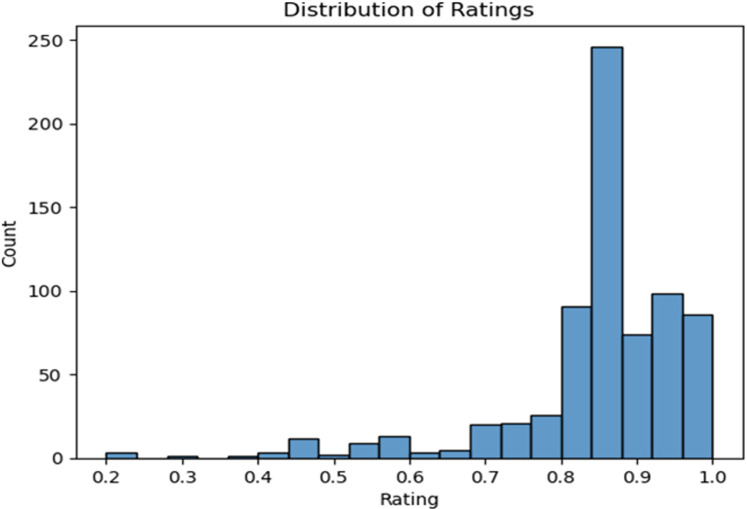
Distribution of App Ratings.

The histogram in Fig 2, reveals a distribution of ratings spanning 0.2 to 1.0. Most apps (246) are clustered in the 0.84-0.88 range, indicating positive user feedback. Notably, 86 apps enjoy near-perfect scores between 0.96 and 1.0. In contrast, a few apps (3) have ratings as low as 0.2. There’s a noticeable dip in app frequency in the mid-rating bracket of 0.56 to 0.72, suggesting users often have clear opinions on these apps—either highly favourable or not. This analysis serves as a guidepost to understand user preferences and identify areas for enhancement.

#### 2.3.3. Total reviews by category.

**Fig 3:**
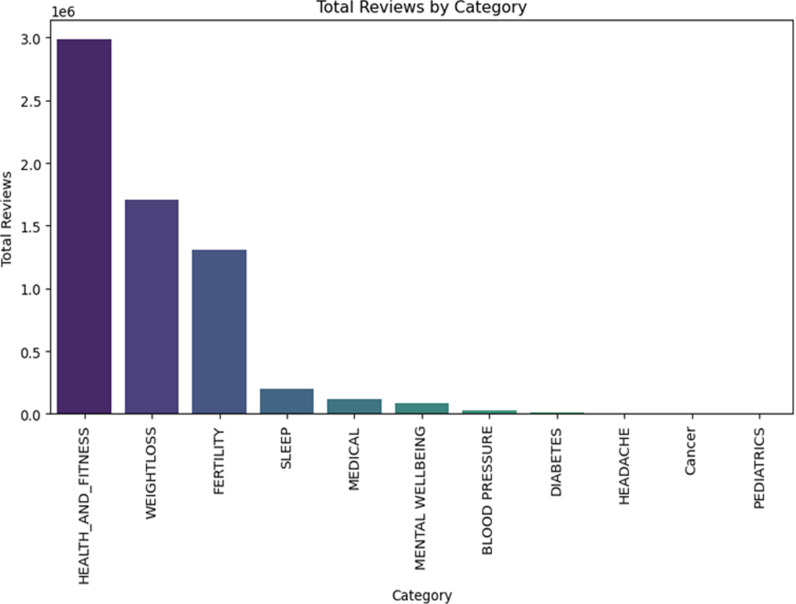
Analysis of Total Reviews for each App Category.

The bar chart in Fig 3, detailing reviews by category highlights user engagement trends across different health apps. Dominating the chart, the ‘Health_and_fitness’ category garners an impressive 2,990,013.4 reviews, indicating not only its popularity but also its pivotal role in users’ routines. ‘Weightloss’ follows with a significant 1,706,068.4 reviews, emphasising the digital world’s increasing focus on health and wellness. In contrast, despite their abundance, ‘Medical’ apps gather a mere 114,196.4 reviews, hinting that users might prioritise expert endorsements over peer feedback for such apps. Meanwhile, specialised categories like ‘Headache’, ‘Cancer’, and ‘Paediatrics’ accumulate fewer reviews, reflecting their niche nature.

#### 2.3.4. Total installs by category.

**Fig 4:**
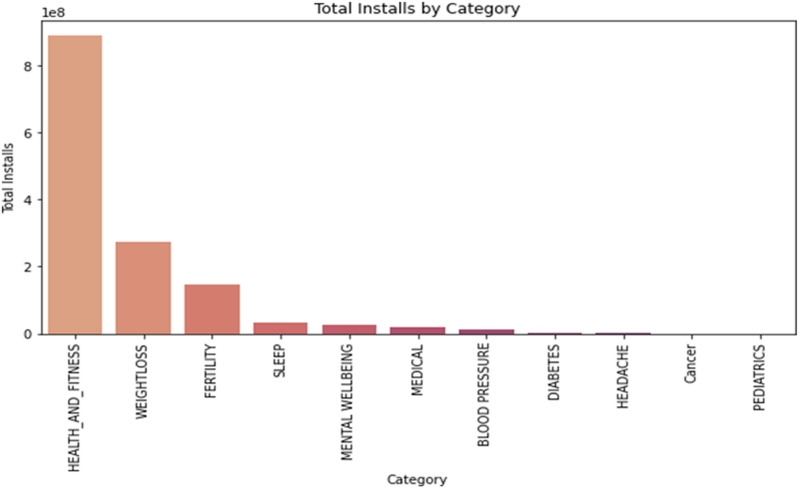
Analysis of Total Installs for each App Category.

The bar chart in Fig 4, illustrates the dominating the scene, ‘Health_and_fitness’ apps amass a staggering 890,877,292 installs, underscoring the growing trend towards health-conscious digital tools. Following closely, the ‘Weightloss’ category registers 274,658,010 installs, reflecting the global emphasis on weight management. ‘Fertility’ and ‘Sleep’ categories also exhibit robust install numbers. However, the ‘Medical’ category lags, suggesting that users might lean towards trusted medical sources over popular app choices when it comes to critical health matters.

#### 2.3.5. Distribution of app ratings.

**Fig 5:**
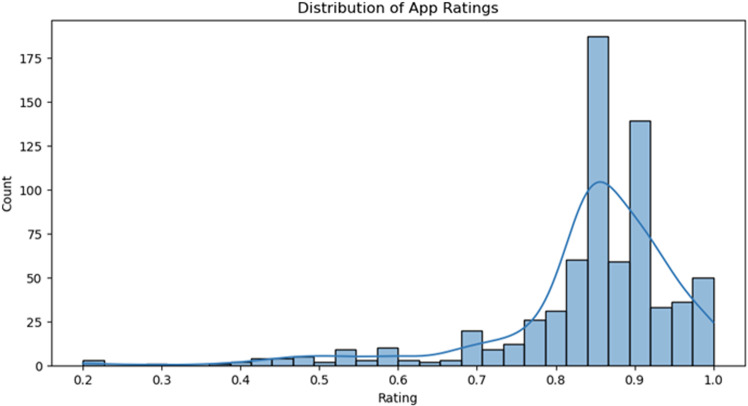
Count of mHealth App Ratings.

The ‘Distribution of App Ratings’ graph in Fig 5, depicts a positive skew, signalling that most health apps have received favourable ratings. A significant concentration of ratings falls between 0.84 and 0.87, with 187 apps residing in this range, reflecting general user satisfaction. Another dense cluster is noticeable between 0.893 and 0.920, housing 139 apps, further reinforcing the positive reception of many health apps. On the flip side, ratings beneath 0.5 are rare, indicating very few apps are poorly perceived by users. The overlaying Kernel Density Estimation (KDE) curve emphasises the peaks around the higher ratings, showcasing the overall user contentment with health apps.

#### 2.3.6. Ratings per categories.

**Fig 6:**
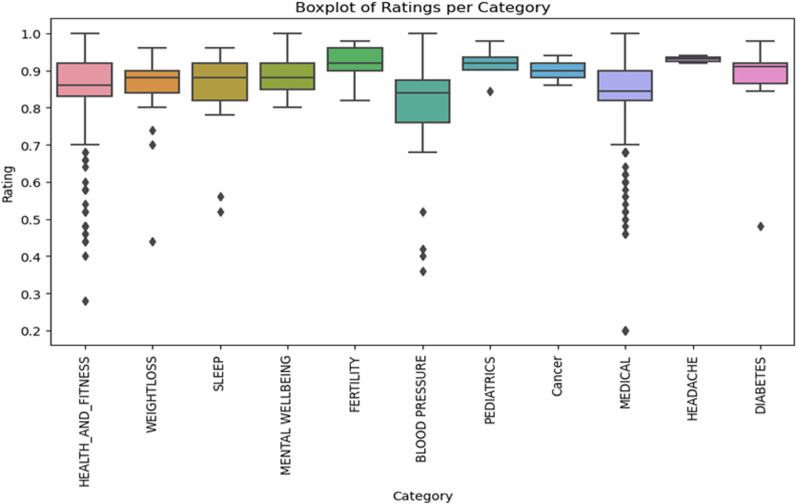
Comparison of user ratings across the app categories.

The ‘Boxplot of Ratings per Category’ in Fig 6, offers insights into user ratings across various health app categories. The interquartile range (IQR) is represented by a box with the median shown as a line within. Categories like ‘Medical’ and ‘Health_and_fitness’ exhibit wide rating variations, while ‘Fertility’ and ‘Headache’ have more consistent feedback. ‘Headache’, ‘Fertility’, and ‘Paediatrics’ tend to have higher median ratings, suggesting user satisfaction. By constrast, the ‘Medical’ category has some outliers, with ratings going as low as 0.20. Average ratings for ‘Cancer’, ‘Fertility’, and ‘Mental Wellbeing’ are primarily above 0.88, while ‘Blood Pressure’ and ‘Sleep’ hover around 0.80. Most categories have apps rated at a commendable 1.00, highlighting their excellence.

#### 2.3.7. Heatmap of correlation matrix.

**Fig 7:**
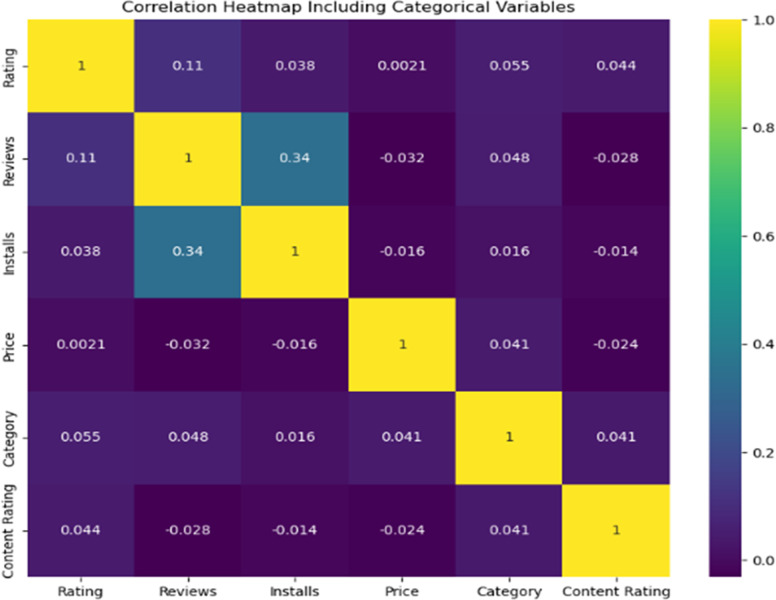
Heatmap showcasing the correlation of numerical and categorical variables.

The heatmap in Fig 7, including Categorical Variables’, visualises linear relationships between several app-related variables, considering label-encoded features ‘Category’ and ‘Content Rating’.

The data reveals a moderate positive correlation of 0.34 between ‘Reviews’ and ‘Installs’, indicating that apps with more installs typically receive more reviews. There’s a mild correlation of 0.11 between ‘Rating’ and ‘Reviews’, suggesting that better-rated apps might receive slightly more reviews. When considering the label-encoded ‘Category’, its correlation with ‘Rating’ is 0.06 and with ‘Reviews’ is 0.05, suggesting only a marginal influence. ‘Content Rating’ predominantly exhibits negligible associations yet shows a slight trend with ‘Rating’ at 0.04. The ‘Price’ variable, on the other hand, displays a lower association with ‘Category’ at 0.04.

### 2.4. Feature engineering

The dataset required extensive preprocessing before modelling. Key categorical variables like ‘Content Rating’, ‘Category’, and ‘is_unrated’ were One-Hot Encoded, resulting in expanded columns representing each unique category. Numerical features were standardised to have a mean of zero and a standard deviation of one, in favour of features like ‘Rating’, ‘Reviews’, and ‘Price’. A unique feature, ‘Rating_Reviews’, was introduced, capturing the cumulative influence of ratings and reviews. This feature was calculated by multiplying the normalized values of ratings and reviews to create a single metric that represents the overall user feedback for each app. The theoretical basis for this combination lies in the fact that both ratings and reviews contribute to the perceived quality and popularity of an app, and their combined effect can provide a more comprehensive measure of user satisfaction. This approach aligns with research that emphasizes the importance of integrating multiple dimensions of user feedback to accurately assess app performance and user engagement [10].

The choice of ‘Category’ as the target variable aimed to contextualise health apps, ensuring precision in recommendations. After one-hot encoding, ‘Category’ columns were streamlined into a single column by identifying the dominant category per row, simplifying the dataset and making it more suitable for modelling. Additionally, non-essential columns such as ‘App’ and ‘Size’ were deemed non-influential for the predictive modeling and were dropped. This decision was driven by the need to focus on the most relevant features that impact the performance of the recommendation system. Rigorous quality checks were performed to flag and handle any negative values in the dataset, ensuring the integrity of the data.

Before proceeding to modelling, a clear demarcation was made between features and the target variable. The extensive dataset, post one-hot encoding, necessitated a focused feature selection process. The “SelectKBest” object method with “mutual info classification” was used as a scoring function to assess the relationship between each feature and the target variable, leading to the identification of 10 essential features considered instrumental for the modelling phase. The decision to drop certain columns and ensure data quality was driven by the nature of the data within these columns and the potential influence on modelling algorithms. The culmination of these steps set the stage for effective model implementation, ensuring that the dataset was both robust and streamlined for the subsequent analysis.

#### 2.4.1. Splitting the data for model training and testing.

To evaluate the performance of the machine learning models, the dataset was divided into training and test sets. This division ensures that the model’s performance can be assessed on unseen data, providing a realistic measure of its generalization capability. The dataset was split into 80% for training and 20% for testing. This proportion is commonly used in machine learning to balance between having enough data to train the model and enough data to reliably test its performance. Research by Kohavi (1995) [11] suggests that this 80-20 split is effective for cross-validation and provides a representative distribution of features in both sets, reducing the risk of overfitting and ensuring that the model’s performance is not biased by the training data.

The training set was used to fit the model, allowing it to learn the underlying patterns and relationships within the data. The test set, kept separate during training, was later used to evaluate the model’s performance. This evaluation ensures that the results reflect the model’s ability to generalize to new, unseen data. Such a methodical approach to feature engineering and data splitting is crucial for training models on robust, well-prepared data and for evaluating their performance in a manner that mirrors real-world applicability.

The theoretical foundation for this practice lies in the concept of statistical learning, where the goal is to create models that not only fit the training data but also perform well on new, unseen data. According to Hastie, Tibshirani, and Friedman (2009) [12], ensuring a model’s generalization capability is fundamental to achieving reliable predictive performance. By splitting the data into training and test sets, we can estimate the model’s generalization error, providing insights into how well the model will perform in practical applications.

## 3. Results and discussion

### 3.1. Model Implementation and Results

In the endeavour to build a robust text classification model, a range of algorithms and techniques were explored. The performance of these models was evaluated on training, validation, and independent test sets to ensure their ability to generalize to new, unseen data. The results of these techniques and models are summarised in the following Table 1:

**Table 1:** Evaluation Metrics of all Models Performed.

Model	Accuracy (%)	Precision (avg)	Recall (avg)	F1-Score (avg)	Remarks
Random Forest Classifier	52	0.48	0.52	0.48	Baseline model with decent performance.
TF-IDF Vectorizer + RF	74	0.79	0.74	0.72	TF-IDF could provide better text representation than raw frequency.
LSTM	67	0.74	0.67	0.68	LSTM is suitable for sequential data like text.
LSTM with L2 Regularization	64	0.71	0.64	0.65	Regularization did not significantly boost performance.
Hybrid CNN- LSTM	49	0.40	0.49	0.41	The combination did not significantly boost performance.
Transfer Learning (BERT)	86.01	–	–	–	Transfer learning with BERT provided a significant performance boost.
Transfer Learning + Hyperparameter Tuning (BERT)	89.51	0.89	0.90	0.89	Further fine- tuning improved the model’s performance.

Random Forest Classifier was initially used as a baseline model, achieving an accuracy of 52% and an F1-Score of 0.48. This ensemble learning method, which constructs multiple decision trees for predictions [13], hinted at potential improvements through feature engineering or hyperparameter tuning. Integrating the TF-IDF Vectorizer, which evaluates word importance in relation to a corpus [14], with the Random Forest improved the accuracy to 74% and an F1-Score to 0.72. The deep learning approach using LSTM networks, designed for sequential data processing [15], yielded an accuracy of 67%. However, incorporating L2 Regularization to counter overfitting [16] slightly reduced the accuracy to 64%. This indicated that while regularization can help mitigate overfitting, it might also affect the model’s ability to capture complex patterns in the data. Lastly, a Hybrid CNN-LSTM model, combining CNNs for feature extraction with LSTMs for sequence capture [10], resulted in an accuracy of 49%. The independent test set evaluation indicated that the model’s performance on unseen data was moderate, highlighting the necessity for further refinement and regularization techniques to enhance generalizability.

Given these results, attention is drawn to the BERT Transfer Learning model due to its state-of-the-art capabilities and adaptability. Its architecture, designed for extensive pre-trained tasks, makes it particularly suited for the dataset at hand, outshining traditional and other deep learning models. The fine-tuning of BERT, supported by meticulous hyperparameter tuning, holds the promise of achieving high-level performance, justifying its selection for deeper exploration.

### 3.2. Transfer Learning (BERT)

Transfer learning, a technique in deep learning, repurposes models trained on one task for related tasks, often yielding impressive results with limited data. A prime example is the BERT model (Bidirectional Encoder Representations from Transformers) by Devlin et al. (2018) [17]. Pre-trained on extensive text corpora, BERT captures complex language patterns and, when fine-tuned, showcases state-of-the-art performance in NLP. Unlike traditional recurrent layers, BERT utilizes the Transformer architecture from Vaswani et al. (2023) [18] which processes sequences simultaneously through attention mechanisms. What sets BERT apart is its bidirectional training, where it predicts missing words using context from both preceding and following words. This approach deepens its semantic understanding, leading to a remarkable accuracy of 86.01% for the given task.

**Fig 8:**
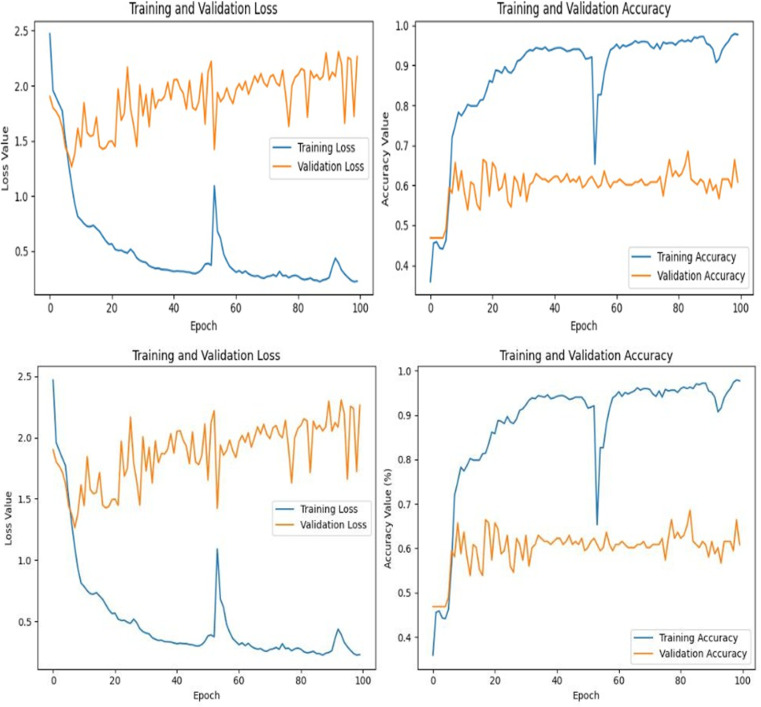
Train and Validation Loss/Accuracy Plot.

The initial plots in Fig 8, showcase the performance of the pre- trained BERT model over multiple epochs. As the epochs increase, Training Loss consistently decreases while Validation Loss begins to increase, suggesting overfitting. This implies the model may be overly adjusting to the training data, risking its generalisation capabilities. Concurrently, the Training and Validation accuracies rise steadily, albeit with a dip around the 50th epoch, further confirming the overfitting suspicion. The training and validation loss and accuracy curves (Fig 8) demonstrated consistent improvement over 50 epochs. The validation accuracy approached the training accuracy, suggesting a well-generalized model with minimal overfitting. Despite this setback, the model concludes with commendable accuracy.

The subsequent graphs, representing the hyper-tuned model, echo the patterns seen in the pre-trained model. The overfitting indications are consistent, but notably, the hyper-tuned model concludes with a marginally superior accuracy, highlighting the benefits of hyperparameter tuning in model optimisation.

### 3.3. Transfer Learning +  Hyperparameter Tuning (BERT)

Further refining the approach, Hyperparameter Tuning was employed on the BERT model. Hyperparameters, parameters not learned from the training process, play a pivotal role in model training [19]. However, the efficacy of a model is not solely based on its architecture. The training process, and especially the hyperparameters used during this process, play a critical role. Hyperparameters can be thought of as the ‘settings’ or ‘knobs’ for the training process. They are not updated during training and need to be specified before the training begins. The learning rate, for instance, determines the size of steps the model should take towards minimizing the error. If it’s too large, the model might overshoot and never converge. If it’s too small, the model might take an excessive amount of time to train or might get stuck in a local minimum.

The epsilon hyperparameter in the Adam optimizer ensures that the model doesn’t encounter division by zero errors, ensuring numerical stability. Tuning these hyperparameters is essential. Even the best models can perform poorly with non-optimal hyperparameters.

BERT’s bidirectional nature sets it apart from many traditional models. Unlike models like LSTMs which read text in a unidirectional manner (either left-to-right or right- to-left), BERT considers both directions. This bidirectionality ensures that for every word, its entire context (words to its left and right) is considered. This is a revolutionary step, ensuring a profound understanding of the context in which words appear.

Central to BERT is the Transformer architecture, an innovation by Vaswani et al. (2023) [18]. The architecture is anchored on attention mechanisms, specifically the self- attention mechanism. This mechanism allows the model to weigh the significance of different parts of the input data. The result is that the model can dynamically focus on more pertinent parts of the input for any given output part. Mathematically, the attention mechanism is formulated as:


$$Attention\left(Q,K,V\right)=softmax\left(\frac{QK^{T}}{\sqrt{d_{k}}}\right)V$$

In this formula, Q stands for the Query matrix, K stands for the Key matrix, V stands for the Value matrix, d_k_ is the dimensionality of the key vectors and the SoftMax function is used to transform the similarity scores (from the dot product of Q and K) into attention weights. The weights in the attention mechanism allow the model to concentrate on different words in the input sequence variably, offering a nuanced understanding of context.

The self-attention mechanism, play a pivotal role. This mechanism leverages the matrices: Q, K and V. The attention scores are computed by taking the dot product of Q and K, which then gets divided by the square root of the depth (d_k_) to stabilize the scores. The SoftMax function subsequently converts these raw scores into a probability distribution, emphasising tokens with higher relevance and diminishing the less relevant ones. This ensures the model dynamically focuses on the most pertinent parts of the input, providing a context-aware output.

### 3.4. Hyperparameter Tuning

Hyperparameter tuning is essential in machine learning to fine-tune the learning process. Unlike model parameters, which are data-driven, hyperparameters are predetermined and can profoundly influence learning. For instance, in the Adam optimizer, the learning rate determines step sizes towards minimizing loss, while epsilon ensures numerical stability [20]. Utilizing the ‘kerastuner’ library, the Hyperband tuner method was employed, combining random search and early stopping for optimal hyperparameter optimisation [21]. To evaluate the generalization ability of the hyper-tuned BERT model, we tested it on the independent test set. The effectiveness of this method is evident in the 90% accuracy achieved. By integrating BERT, a powerful model, with diligent hyperparameter tuning, the model’s performance soared, resulting in an accuracy of approximately 90% and an F1-Score of 0.89. This high accuracy on the test set demonstrates the model’s strong ability to generalize to new, unseen data, validating its robustness and efficiency beyond the training and validation phases. It highlights the synergy between transfer learning and rigorous training optimisation in reaching cutting-edge results in NLP tasks.

**Fig 9:**
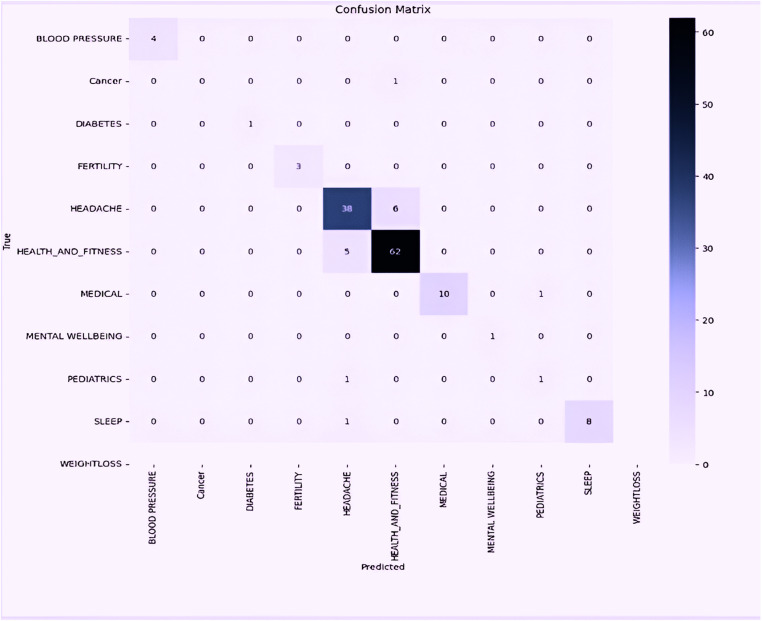
Confusion Matrix of the Hypermodel.

The provided confusion matrix for the hypermodel in Fig 9, reveals that it excels in predicting the ‘Headache’ and ‘Health and Fitness’ categories, with 38 and 62 accurate classifications respectively. However, a recurring misclassification occurs between these two categories, with ‘Headache’ mistaken for the ‘Health and Fitness’ 6 times, and vice versa 5 times. This suggests that there might be overlapping features or descriptions leading to such confusion. Other categories, like the ‘Blood Pressure’, ‘Diabetes’, ‘Fertility’, ‘Medical’, ‘Paediatrics’, and ‘Sleep’, demonstrate minimal misclassifications, indicating the model’s overall efficacy in distinguishing between most categories. The BERT model’s ability to maintain high performance on the test set underscores its potential for real-world application. This highlights the importance of using advanced architectures and transfer learning to build robust models capable of handling diverse and unseen data.

To conclude the research, a health app recommendation system was designed to cater to the specific needs of users by leveraging similarity metrics. To begin, the system catalogued the range of health categories it had in its database. When a user inputted their requirements, the system quickly matched the query to a pertinent health category using predefined keywords. Upon identifying the health category, it harnessed the power of a pretrained model to generate embeddings for the user’s query and the app descriptions within the chosen category. The system then employed cosine similarity, a mathematical metric used to determine the degree of similarity between two vectors, to rank the apps based on their relevance to the user’s query. This methodology ensured precision, as the system consistently recommended the top five apps that best aligned with the user’s needs. For instance, queries related to sleep returned apps that assist with sleep tracking or offer relaxation sounds, while a pregnancy-related search provided apps geared towards tracking periods, ovulation, and pregnancy stages, emphasising the system’s ability to offer nuanced and contextually relevant suggestions.

## 4. Conclusion and recommendations

The exploration of the mHealth domain illuminated the nuances and intricacies of building a precise health app recommendation system. Traditional models, though foundational, were found to be limited in grasping the subtleties embedded in app descriptions. This necessitated the transition to more sophisticated models, with a transfer learning model (BERT), supplemented with hyperparameter tuning, emerging as the standout performer, achieving an accuracy of approximately 90%. These findings not only validate the efficacy of advanced algorithms in parsing vast datasets but also highlight the potential for such systems to revolutionize user interaction with health applications. The designed system effectively maps user inquiries to a curated list of applications, facilitating an enhanced user experience by prioritizing relevance and personalisation. By addressing the previously identified gap in the market, this research contributes a meaningful tool to assist consumers in navigating the saturated landscape of health apps.

To elevate the efficacy and applicability of the machine learning-based recommendation system for mobile health apps, it is crucial to expand the datasets used. By incorporating a more varied data, the system can achieve greater robustness and relevance. In parallel, broadening the range of health categories recognized by the system is essential. This expansion will not only cater to a more extensive array of health conditions but also enhance user satisfaction by providing more personalised options. Additionally, there is a continuous need to delve into more advanced models and algorithms. Embracing these sophisticated computational tools can substantially refine the system’s predictive precision and its ability to adapt to the evolving landscape of mobile health apps, ensuring that recommendations remain cutting-edge and user-centric.

## 5. Limitations and future work

The study, however, faced challenges rooted in data acquisition and its inherent limitations. The 2018 dataset was not only challenging to procure but also presented limitations in its currency and breadth. This was evident in the initial models’ performance and the restricted illness categories like Cancer, which could have benefited from a more extensive range.

Given the limitations faced, it is recommended that future research endeavours prioritize the collation of more recent datasets, potentially partnering with app stores or health organizations for more robust and comprehensive data. Expanding the illness categories would not only enhance the system’s robustness but also its applicability to a broader user base. Feedback-driven refinements are essential for adapting to the ever-evolving app ecosystems. Addressing the overlapping category definitions and exploring additional advanced models can further refine the recommendation system. Moreover, integrating real- time user feedback mechanisms can provide dynamic insights, allowing for ongoing system optimisation.

For researchers aiming to replicate or extend this study, it is crucial to ensure that any data used complies with GDPR guidelines and other relevant data protection regulations. While our dataset focused solely on app names and statistical information, avoiding personal user data, future datasets must be carefully vetted to protect user privacy. Implementing robust data anonymization techniques and ensuring that data is encrypted during transmission and storage are necessary steps. Clearly communicating to users how their data will be used and obtaining explicit consent before collecting any personal information is also crucial. Providing users with options to opt-in or opt-out of data collection and use will enhance transparency and trust.

To ensure fairness, future models should be regularly audited and tested to identify and mitigate biases. Implementing fairness-aware machine learning techniques will help ensure that recommendations are unbiased and equitable across different user demographics. Developers seeking to implement or adapt this paper, developing strategies to promote digital inclusion - such as providing access to affordable smartphones and internet connectivity in underserved communities - is vital. Collaborating with local organizations to raise awareness and provide training on using health apps effectively can help bridge the digital divide.

For developers, it is advised to design the recommendation system interface to be user-friendly and transparent. Providing explanations for how recommendations are generated and allowing users to provide feedback or ask questions about the recommendations will enhance understandability and user trust. Establishing accountability mechanisms for the recommendation system, including regular performance evaluations and audits, is necessary. Creating channels for users to report any inaccuracies or issues with the recommendations and taking prompt corrective actions will ensure accuracy and responsibility. Lastly, developers should design the recommendation system with accessibility features such as screen readers and voice commands. Ensuring compatibility with a wide range of devices and assistive technologies will accommodate users with disabilities, promoting inclusivity. By addressing these aspects, developers can create a more robust, reliable, and accessible recommendation system, ultimately providing accurate and equitable health app recommendations to a diverse user base.
